# Supplementary material for: Synthetic flow-based cryptomining attack generation through Generative Adversarial Networks
Source: Sci Rep. 2022 Feb 8;12:2091. doi: 10.1038/s41598-022-06057-2 (PMC8825844; doi:10.1038/s41598-022-06057-2)
Supplement: Supplementary file 1 — Supplementary Figures. [file 41598_2022_6057_MOESM1_ESM.pdf]

Synthetic flow-based cryptomining attack generation through Generative Adversarial Networks

Supplementary file S1: Histograms of experiments

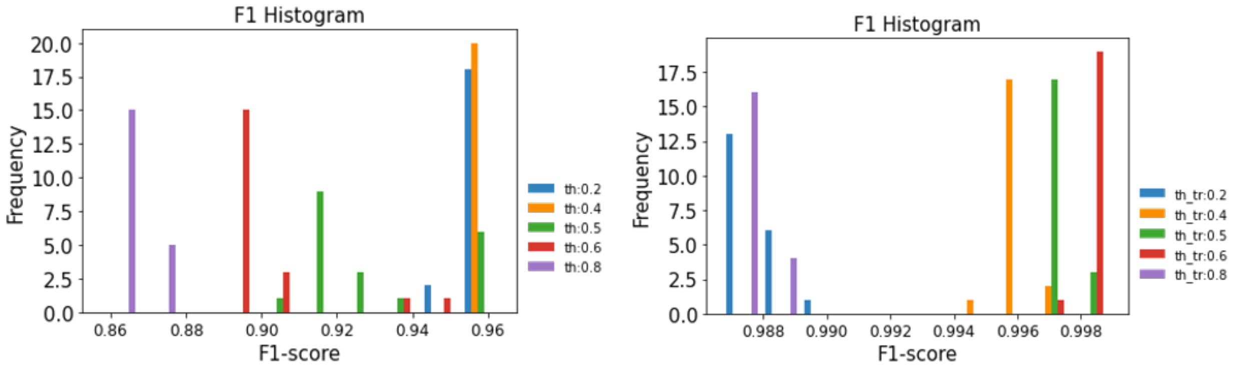

(a) F1-score on testing (left) and training (right) using real data for training and testing (400K/4K distribution).

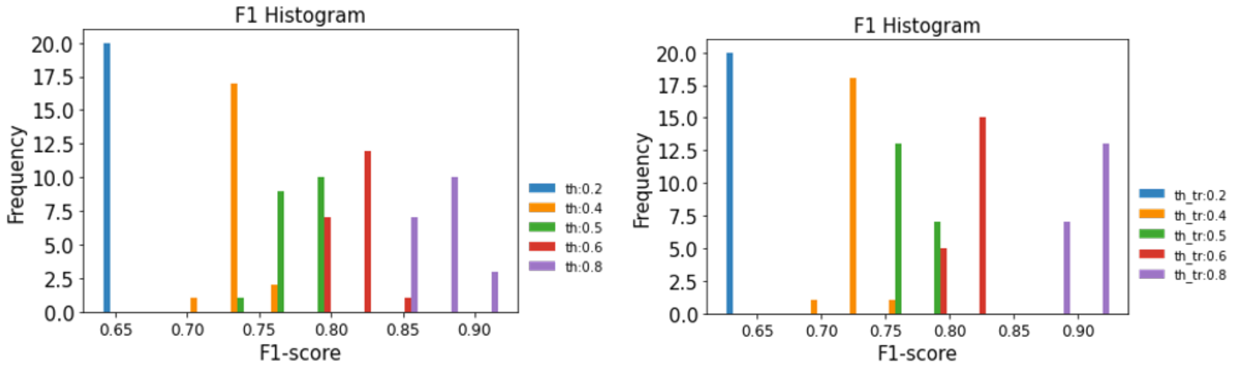

(b) F1-score on testing (left) and training (right) using real data for training and testing (4K/4K distribution).

Figure S1. F_1-score on testing (left) and training (right) using real data with 400K/4K (a) and 4K/4K (b) class distributions for training). Results for decision thresholds of 0.2, 0.4, 0.5, 0.6 and 0.8 are represented.

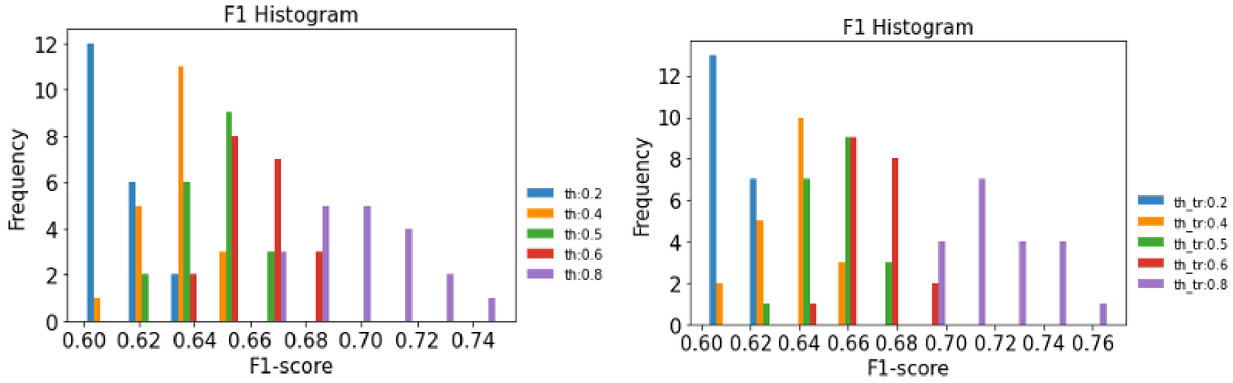

(a) Unbalanced dataset with the original 400K/4K distribution.

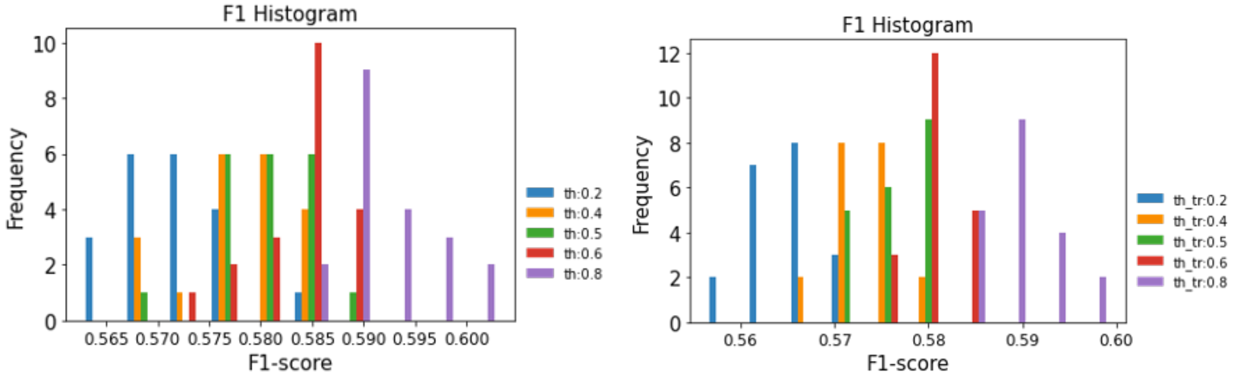

(b) Balanced dataset with 4K/4K distribution.

Figure S2. F_1-score on testing (left) and training (right) using a naïve mean-based generator with unbalanced and balanced datasets for training. Results for decision thresholds of 0.2, 0.4, 0.5, 0.6 and 0.8 are represented.

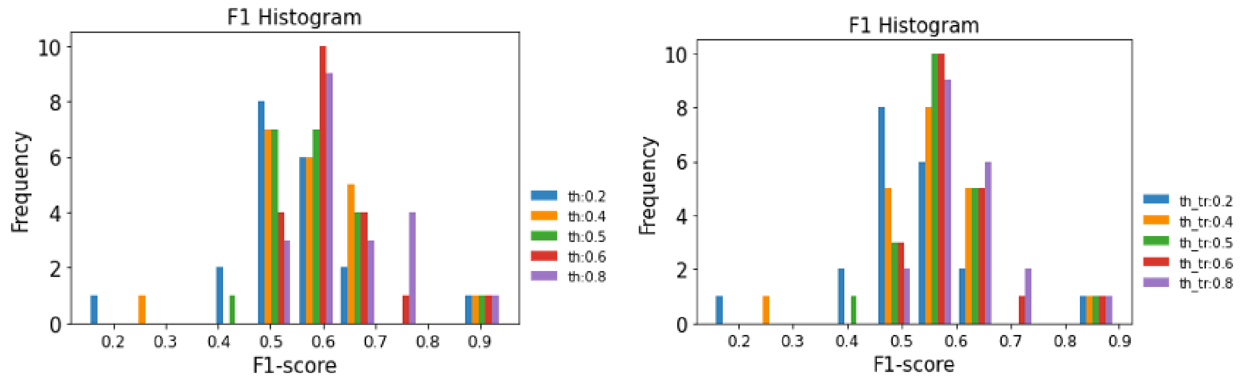

(a) Policy 1). Training with 400K/4K distribution and one generator chosen uniformly at random

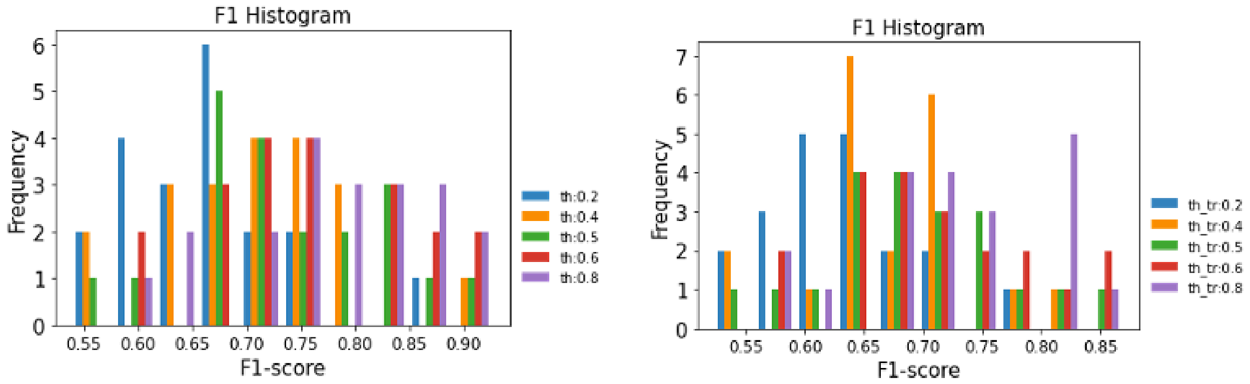

(b) Policy 2). Training with 400K/4K distribution and a mix of two generators is chosen uniformly at random

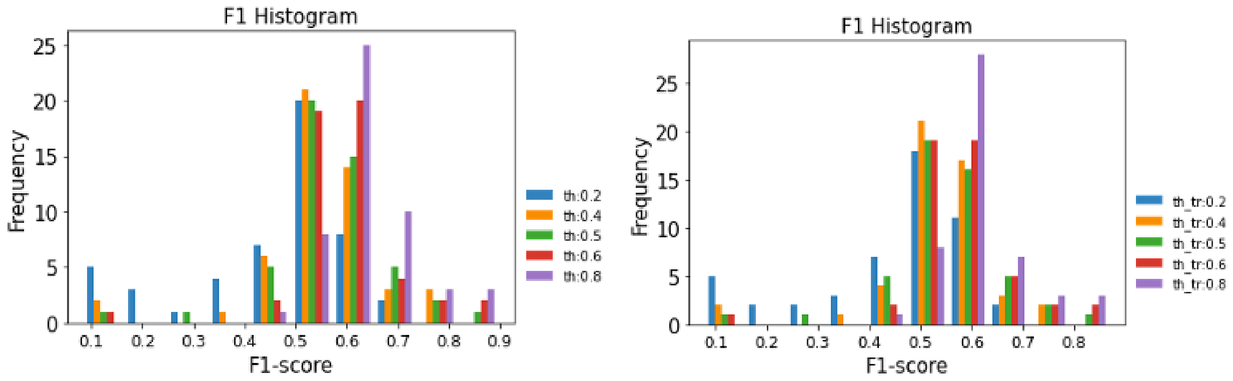

(c) Policy 3). Training with 4K/4K distribution and one generator chosen uniformly at random

Figure S3. F_1-score on testing (left) and training (right) using a standard GAN generator and sampling policies 1), 2) and 3). Results for decision thresholds of 0.2, 0.4, 0.5, 0.6 and 0.8 are represented.

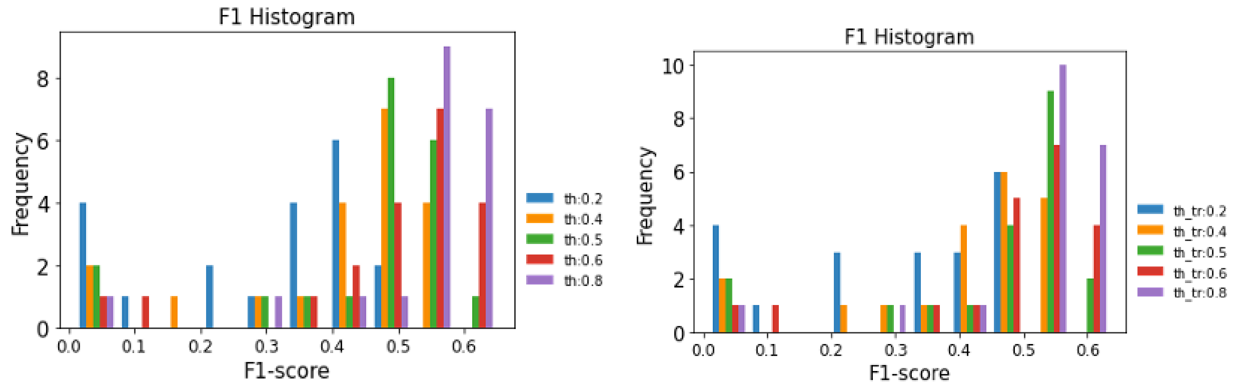

(a) Policy 1). Training with 400K/4K distribution and one generator chosen uniformly at random

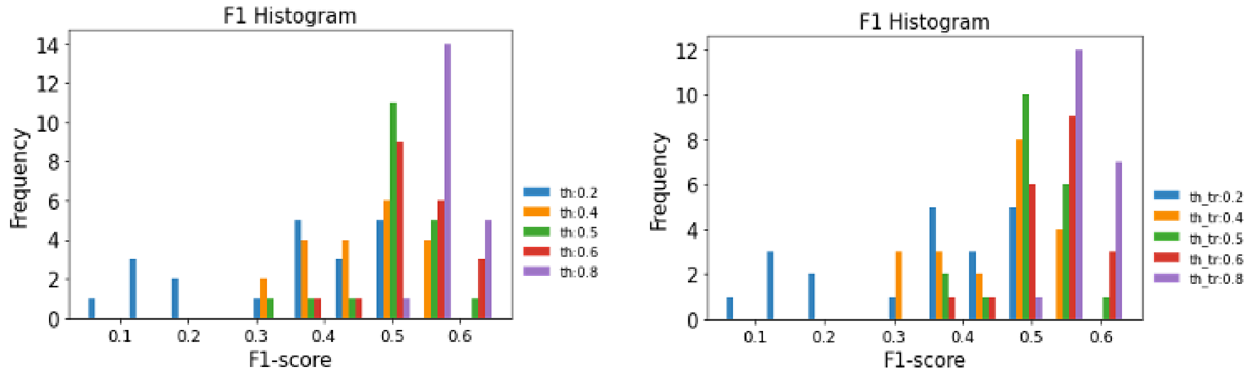

(b) Policy 2). Training with 400K/4K distribution and a mix of two generators is chosen uniformly at random

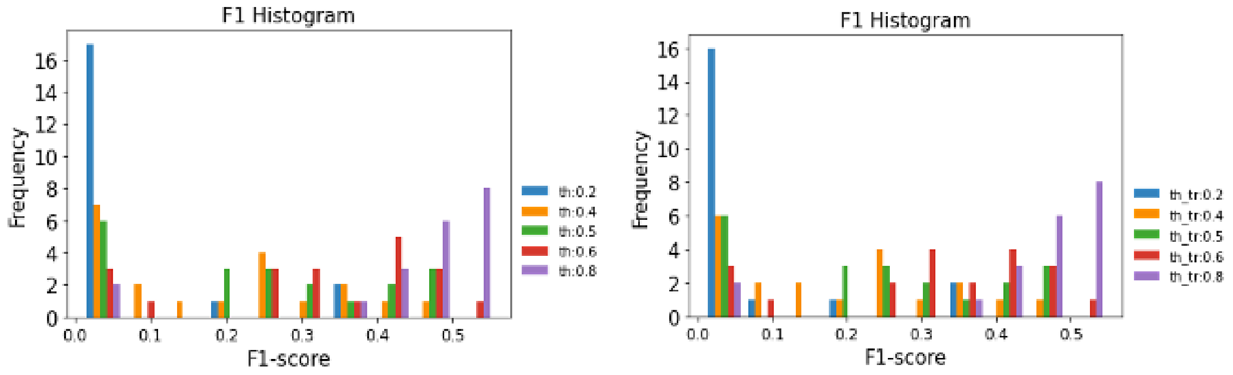

(c) Policy 3). Training with 4K/4K distribution and one generator chosen uniformly at random

Figure S4. F_1-score on testing (left) and training (right) using a generator with custom activation functions at the output and policies 1), 2) and 3). Results for decision thresholds of 0.2, 0.4, 0.5, 0.6 and 0.8 are represented.

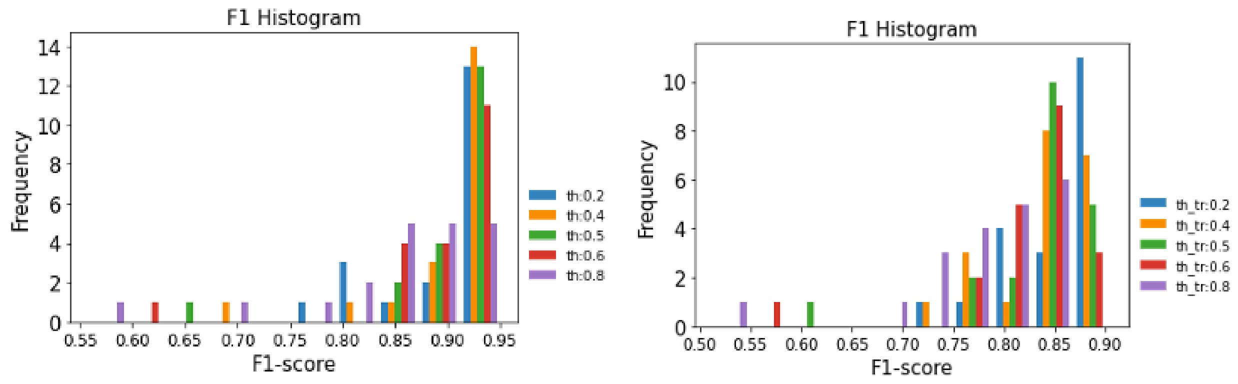

(a) Training with 400K/4K distribution. 1 generator chosen uniformly at random

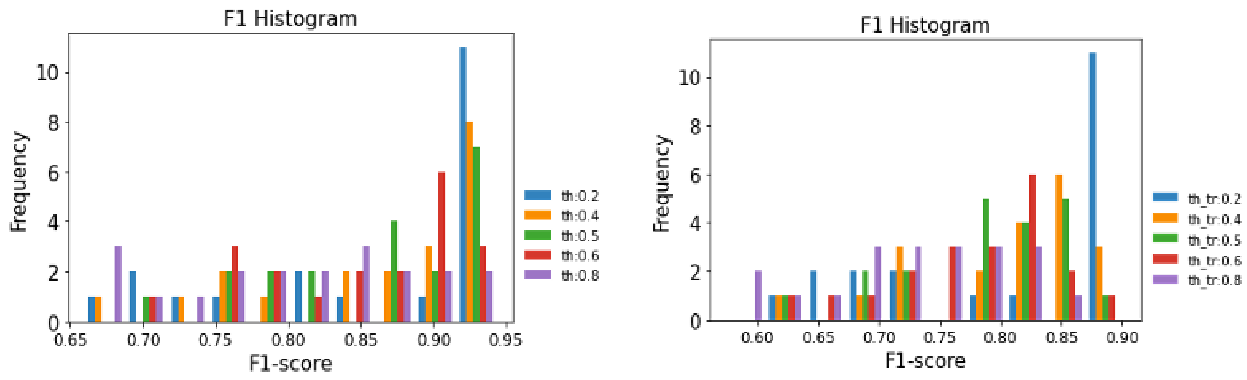

(b) Training with 400K/4K distribution. 1 generator chosen uniformly at random filtering positive values

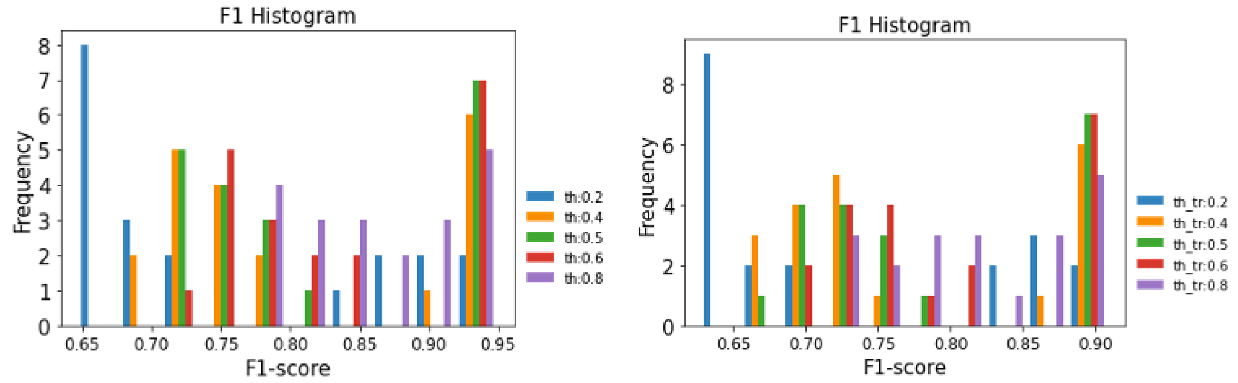

(c) Training with 4K/4K distribution. 1 generator chosen uniformly at random

Figure S5. F_1-score on testing (left) and training (right) with sampling elitism among the top 10 models in training sorted by F_1-score. Results for decision thresholds of 0.2, 0.4, 0.5, 0.6 and 0.8 are represented.

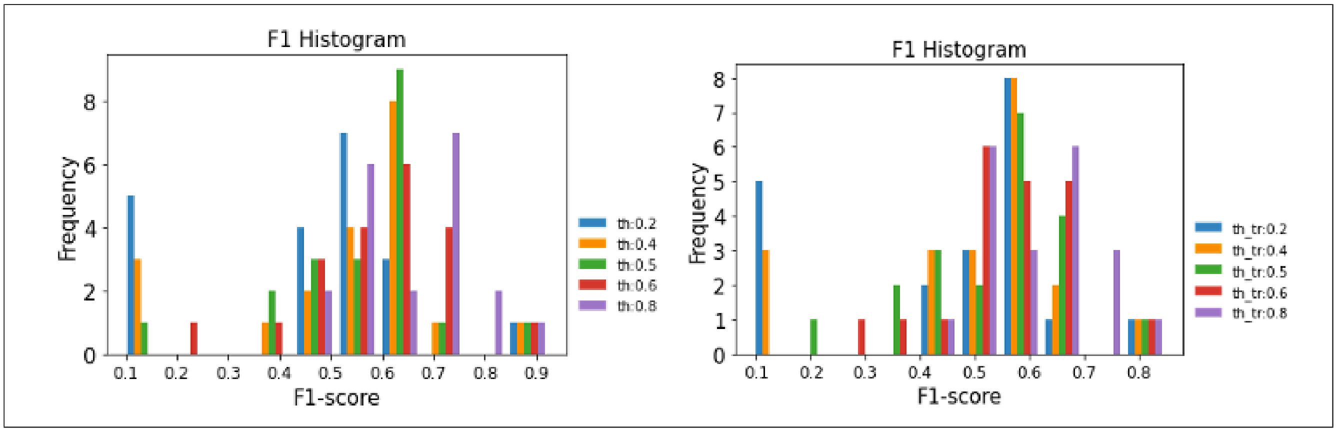

Figure S6. F_1-score on testing (left) and training (right) using the discriminator as a quality assurance filter. Training with 400K/4K distribution. Results for decision thresholds of 0.2, 0.4, 0.5, 0.6 and 0.8 are represented.

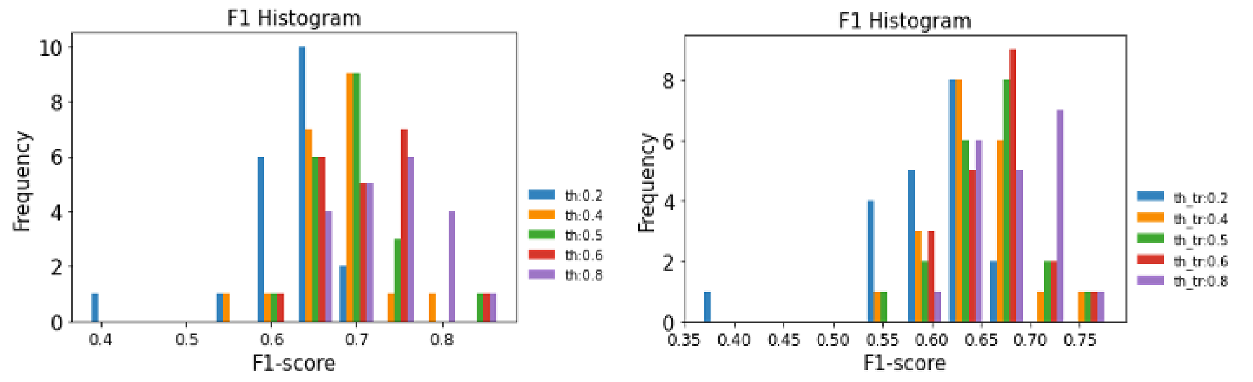

(a) Using top 10 models sorted by L^1 distance

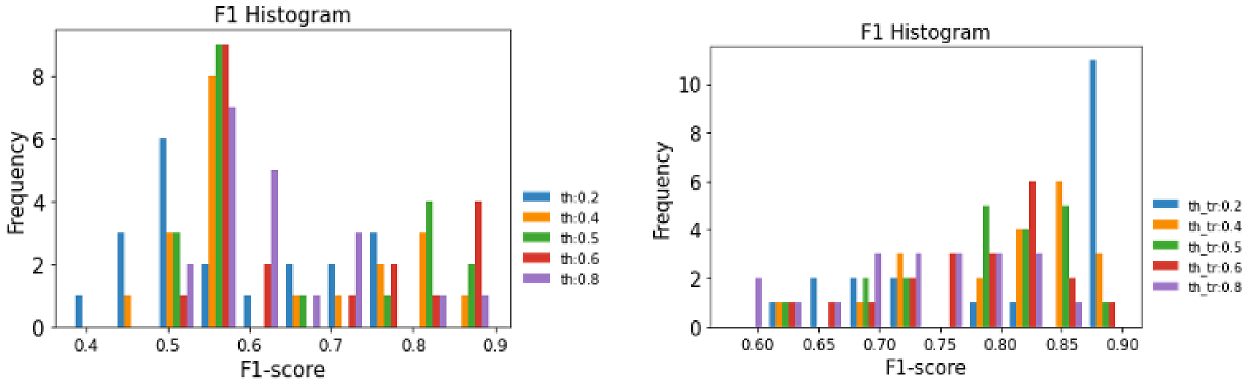

(b) Using top 10 models sorted by Jaccard index

Figure S7. F_1-score on testing (left) and training (right) with sampling elitism using policy 1). Elitism of the top 10 sorted by statistical coefficients . Results for decision thresholds of 0.2, 0.4, 0.5, 0.6 and 0.8 are represented.
